# The Autocrine/Paracrine Loop After Myocardial Stretch: Mineralocorticoid Receptor Activation

**DOI:** 10.2174/1573403X113099990034

**Published:** 2013-08

**Authors:** Irene L Ennis, Ernesto A Aiello, Horacio E Cingolani, Néstor G Pérez

**Affiliations:** Centro de Investigaciones Cardiovasculares, Facultad de Ciencias Médicas, Universidad Nacional de La Plata, Calle 60 y 120, 1900 La Plata, Argentina

**Keywords:** Myocardial stretch, slow force response, Anrep effect, mineralocorticoid receptor, reactive oxygen species, Na^+^/H^+^ exchanger activation.

## Abstract

The stretch of cardiac muscle increases developed force in two phases. The first phase, which occurs rapidly, constitutes the well-known Frank-Starling mechanism and it is generally attributed to enhanced myofilament responsiveness to Ca^2+^. The second phase or slow force response (SFR) occurs gradually and is due to an increase in the calcium transient amplitude as a result of a stretch-triggered autocrine/paracrine mechanism. We previously showed that Ca^2+^ entry through reverse Na^+^/Ca^2+^ exchange underlies the SFR, as the final step of an autocrine/paracrine cascade involving release of angiotensin II/endothelin, and a Na^+^/H^+^ exchanger (NHE-1) activation-mediated rise in Na^+^. In the present review we mainly focus on our three latest contributions to the understanding of this signalling pathway triggered by myocardial stretch: 1) The finding that an increased production of reactive oxygen species (ROS) from mitochondrial origin is critical in the activation of the NHE-1 and therefore in the genesis of the SFR; 2) the demonstration of a key role played by the transactivation of the epidermal growth factor receptor; and 3) the involvement of mineralocorticoid receptors (MR) activation in the stretch-triggered cascade leading to the SFR. Among these novel contributions, the critical role played by the MR is perhaps the most important one. This finding may conceivably provide a mechanistic explanation to the recently discovered strikingly beneficial effects of MR antagonism in humans with cardiac hypertrophy and failure.

## INTRODUCTION

**Fig. (1):**
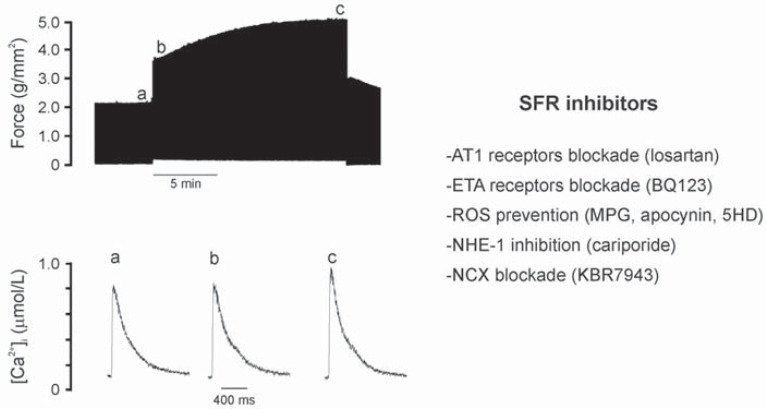
Representative recordings of the contractile response to stretch of an isolated papillary muscle. The first increase in force (from “a” to “b”, top) occurs without changes in the Ca^2+^ transient (“a” to “b”, bottom) while the SFR (from “b” to “c”, top) is due to an increase in the amplitude of the Ca^2+^ transient (“b” to “c”, bottom). Modified from Cingolani *et al*. [68] with permission.

**Fig. (2):**
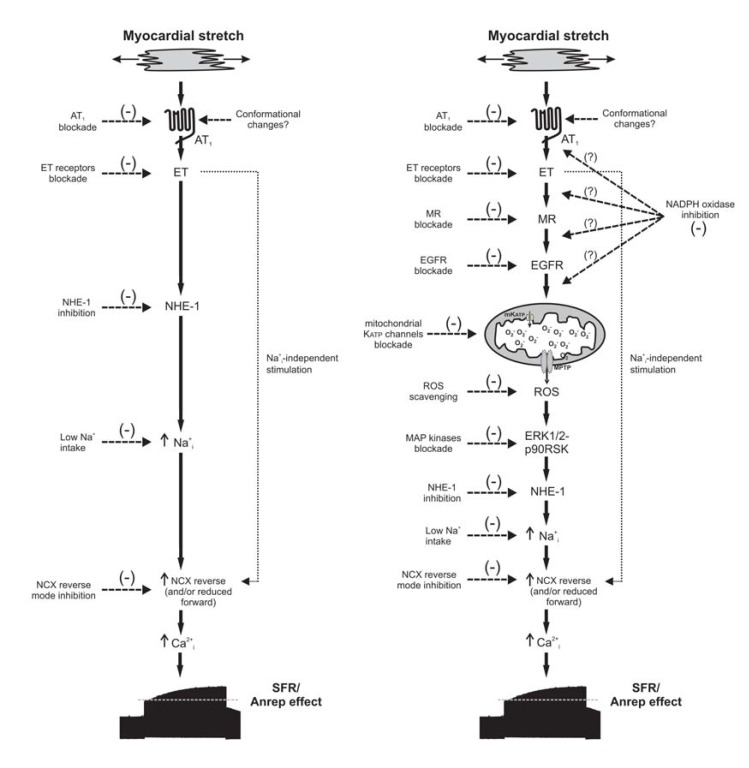
Schematic representation of the chain of events triggered by myocardial stretch that leads to the SFR and probably to cardiac hypertrophy and failure. The left panel depicts the state of knowledge on this subject when we wrote the first version of this review in 2007. On the right panel we present the recent advances in this particular field.

**Fig. (3):**
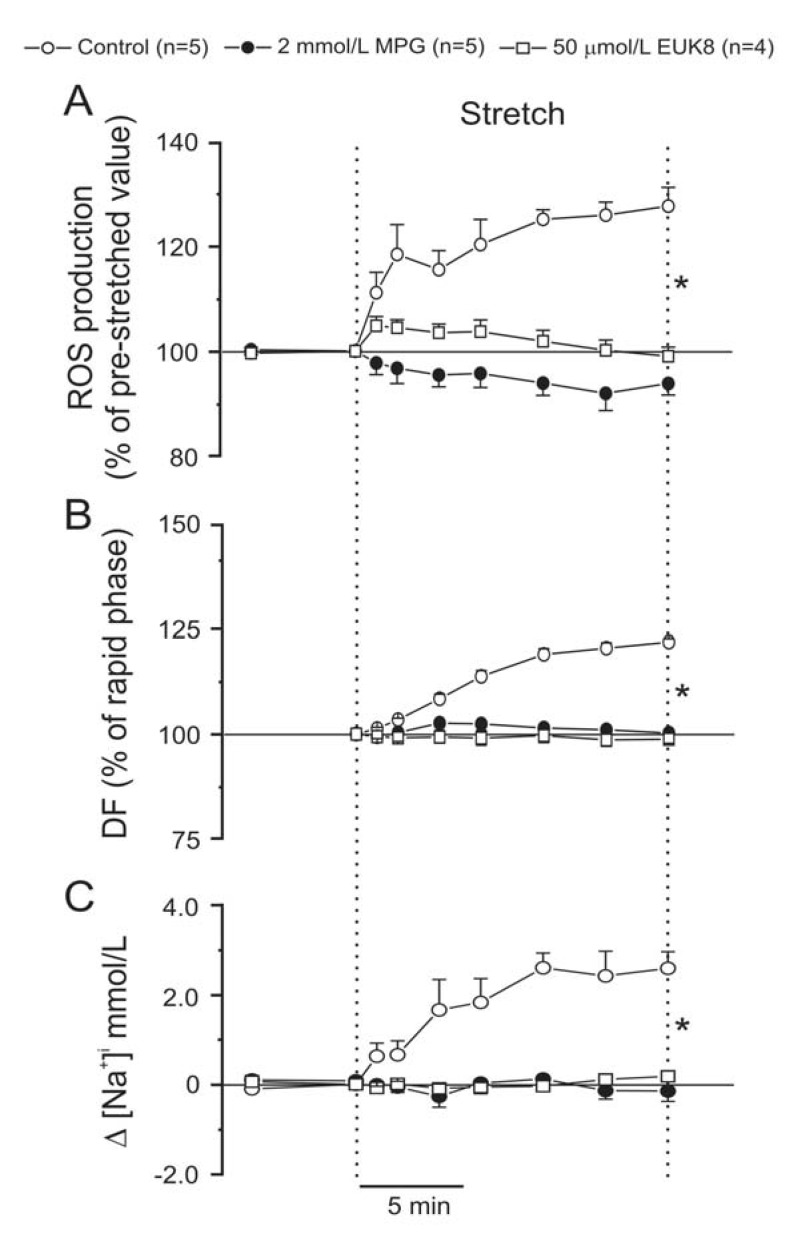
**A.** Myocardial stretch increased intracellular ROS by ~30 % above the baseline level, effect that was cancelled by the ROS scavengers N-(2-mercaptopropionyl)-glycine (MPG) and EUK8. **B.** MPG and EUK8 also cancelled the SFR (expressed as percent of the initial rapid phase). **C.** Furthermore, ROS scavenging also blunted the stretch-induced increase in [Na^+^]_i_. * indicates P < 0.05 control vs. MPG and EUK8. Modified from Caldiz *et al*. [3] with permission.

The stretch of cardiac muscle increases developed force in two phases (Fig. **1**). The first phase, which occurs rapidly, constitutes the well-known Frank-Starling mechanism and it is generally attributed to enhanced myofilament responsiveness to calcium. The second phase or slow force response (SFR) occurs gradually and is due to an increase in the calcium transient amplitude as a result of a stretch-triggered autocrine/paracrine mechanism. The SFR was proposed to be the *in vitro* equivalent to the Anrep phenomenon and its genesis is still under debate. This is an area of research in which we have been working for almost the last 15 years, unveiling much of the components of the signalling pathway triggered by stretch that leads to the slow increase in contractility and very probably to cardiac hypertrophy development [For review see [1]]. A schematic summary of the autocrine/paracrine chain of events proposed by us to be triggered by myocardial stretch at the time when we wrote the first version of this review is presented in the left panel of (Fig. **2**). Briefly, it was as follows: **1.** Stretch-triggered release of A2/activation of AT1 receptor (AT1-R), **2.** Release/formation of endothelin (ET), **3.** NHE-1 hyperactivity, 4**.** Increase in intracellular Na^+^ concentration, and **5.** Increase in Ca^2+^ transient amplitude through the Na^+^/Ca^2+^ exchanger (NCX). In the right panel of this figure we present the recent advances in this particular field. Our three latest contributions to the understanding of this signalling pathway triggered by myocardial stretch are: 

***1) The finding that an increased production of reactive oxygen species (ROS) from mitochondrial origin is critical in the genesis of the SFR:*** In 2006 our group established in isolated cat cardiomyocytes that A2, in a concentration that well resembles the physiologic one, increases sarcomere shortening entirely through an autocrine crosstalk with endogenous ET-1. Interestingly, this effect was not only accompanied by a rise in mitochondrial ROS production but also inhibited by the prevention of oxidative stress [2]. Since we had evidence that A2/AT1-R activation initiates the signalling pathway leading to the SFR, we hypothesized that an increase in ROS production could be a step in this signalling cascade. We certainly found that the SFR was accompanied by a ∼30% increased in ROS which promoted NHE-1 activation (Fig. **3**). Supporting the notion of ROS-mediated NHE-1 activation, we found that stretch stimulated the redox-sensitive kinase cascade of the ERK1/2 and p90^RSK^ increasing its level of phosphorylation, effect that was cancelled by AT1-R blockade with losartan. Moreover, scavenging the A2-induced ROS or inhibiting its formation prevented the development of the SFR [3]. We also demonstrated that these ROS were from mitochondrial origin but induced by a small amount of NADPH oxidase-derived ROS [3]. These results were in line with previous reports describing the so-called “ROS-induced ROS-release” phenomenon in which NADPH oxidase-dependent O_2_˙ production triggers the opening of mK_ATP_ channels, inducing mitochondrial depolarization and subsequent mitochondrial ROS generation [4-6].

***2) The demonstration of a key role played by the transactivation of the epidermal growth factor receptor (EGFR):*** The finding by Sadoshima’s group that A2, probably the most widely accepted hypertrophic agent, failed to induce cardiac hypertrophy in transgenic mice overexpressing a mutant AT1 receptor lacking EGFR transactivation in the myocardium [7], lead us to speculate that the SFR, as a result of the myocardial stretch-triggered signalling pathway, would be probably abolished by preventing EGFR transactivation. This speculation was based on the concept proposed by us that the SFR is the mechanical counterpart of the chain of intracellular signals that leads to cardiac hypertrophy development. Among the experimental evidence that support this last concept we should considered: **2.a.** The demonstration that endogenous A2 is the initial mediator of cardiomyocytes hypertrophy after mechanical stretch and that the addition of the surrounding medium of stretched myocytes promoted hypertrophy in non-stretched cardiomyocytes [8]; and **2.b**. The SFR is inhibited by the selective blockade of AT1 receptors [9]. 

**Fig. (4):**
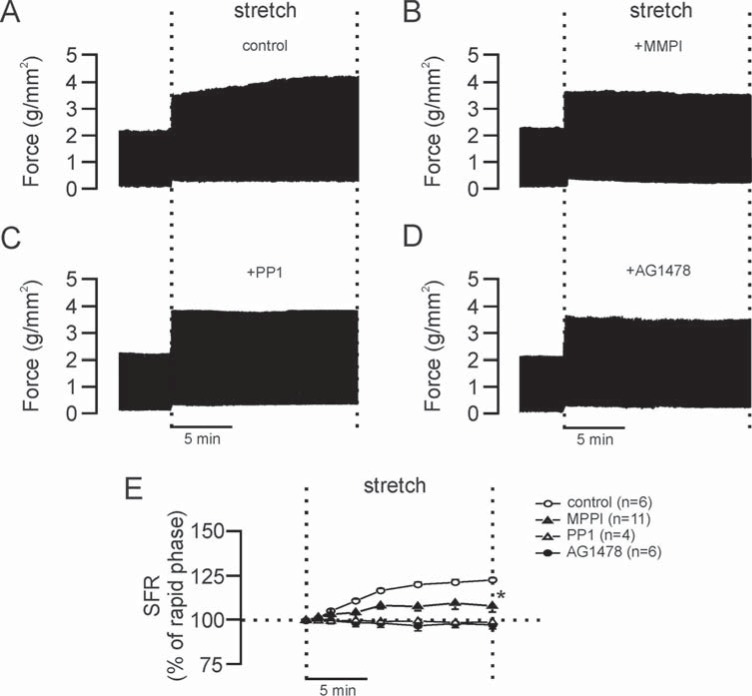
SFR and EGFR transactivation. **Panel A** shows a typical force record from a cat papillary muscle subjected to stretch where it can be appreciated the biphasic response. **Panels B-D**, same as "A" but from muscles pretreated with the matrix metalloproteinase inhibitor (MMPI, "B"), the Src kinase inhibitor (PP1, "C") or the EGFR blocker (AG1478, "D"), interventions that cancel EGFR transactivation. As it can be appreciated, all these pharmacological maneuvers prevented the development of the SFR to stretch. **Panel E** shows the averaged results obtained under the different experimental conditions expressed as percent of the initial rapid phase. * indicates P<0.05 control curve vs. others (2-way ANOVA). Reproduced from Villa Abrille *et al*. [10] with permission.

As it can be appreciated in (Fig. **4**) the development of the SFR was blunted by different interventions that prevent EGFR transactivation, such as inhibition of matrix metalloproteinases, inhibition of Src kinase or the specific blockade of the EGFR, [10]. 

***3) The involvement of the mineralocorticoid receptor (MR) in the stretch-triggered cascade:*** Two lines of previously existing evidence induced us to explore MR activation as a critical step in the signalling cascade leading to the SFR development. **3.a. **The link between A2/AT1 receptor and the MR is an accepted fact [11-13]. **3.b**. The fact that EGFR transactivation can be triggered by MR activation [11, 14, 15], and that MR-dependent increase of EGFR mRNA or protein expression has been reported [16, 17].

**Fig. (5):**
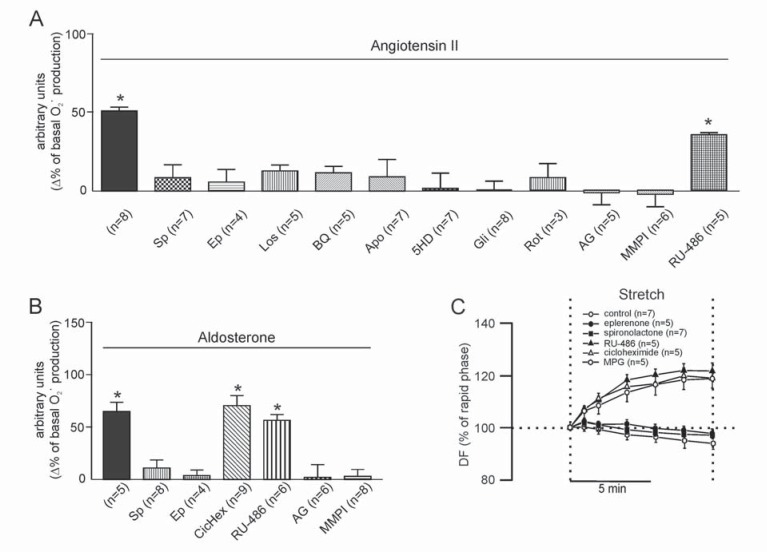
Superoxide anion production induced by angiotensin II (A2). **Panel A:** mineralocorticoid receptor (MR) blockade with spirolactone (Sp, 10 µmol/L) or eplerenone (Ep, 10 µmol/L) abrogated the effect of 1 nmol/L A2 on the basal rate of O_2_˙ production. This effect was also blunted by the AT1 and ET_A_ receptor antagonists losartan (Los, 1 µmol/L) and BQ123 (BQ, 10 µmol/L), respectively, and by NADPH oxidase inhibition with apocynin (Apo, 300 µmol/L), by targeting mitochondria with 5HD (100 µmol/L), glibenclamide (Gli, 50 µmol/L), or rotenone (Rot, 10 µmol/L), and by preventing EGFR activation either by EGFR blockade with AG1478 (AG, 1 µmol/L) or by inhibiting the metalloproteinase involved in EGFR transactivation with MMPI (3 µmol/L). Glucocorticoid receptor inhibition with Ru-486 (10 µmol/L) did not influence the effect of A2. *, *p* < 0.05 vs. basal O_2_˙ production. **Panel B:** The effect of aldosterone (ALD) at a concentration (10 nmol/L) that mimicked the effect of A2 on the basal rate of O_2_˙ production was suppressed by spirolactone (Sp) and eplerenone (Ep), but not by the glucocorticoid receptor inhibitor Ru-486 or by preventing protein synthesis with cycloheximide (CicHex, 7 mmol/L). This demonstrates that MR activation has nongenomic consequences and excludes the possibility of glucocorticoid receptor activation. On the other hand, as shown for A2, the ALD-mediated increase in ROS formation was prevented impeding EGFR activation (AG and MMPI). This suggests that transactivation occurs in the direction of activated MR to EGFR, and that metalloproteinase activation downstream of MR is crucial for EGFR transactivation. *, *p* < 0.05 vs. basal O_2_˙ production. **Panel C:** Averaged results of the SFR expressed as percentages of the initial rapid phase. MR blockade, not only by eplerenone but also by spironolactone, completely suppressed the SFR. However, the SFR was unaffected by the glucocorticoid receptor inhibitor Ru-486 or the protein synthesis inhibitor cycloheximide. Furthermore, the SFR was suppressed by the ROS scavenger MPG, supporting the notion that ROS formation is a key factor in the chain of events leading to the Anrep effect. Modified from Caldiz *et al*. [18] with permission.

Supporting these new lines of evidence we have demonstrated that MR activation is necessary to promote reactive oxygen species formation by a physiological concentration of A2 (1 nmol/L) since the A2-induced production of superoxide anion was abrogated when the MR was antagonized with spironolactone or eplerenone (Fig. **5A**) [18]. This A2 effect was also suppressed by blocking AT1 receptor, ET1 (type A) receptor or EGFR, by inhibiting NADPH oxidase, or by targeting mitochondria; and it was unaffected by glucocorticoid receptor (GR) inhibition [18]. An increase in superoxide anion production promoted by an equipotent dose of aldosterone (ALD, 10 nmol/L) was blocked by spironolactone or eplerenone, by preventing EGFR transactivation, but not after inhibiting GR or protein synthesis, suggesting that it was the consequence of a non-genomic MR effect (Fig. **5B**) [18]. ALD also increased the phosphorylation of the redox-sensitive kinases ERK1/2, p90RSK, and the NHE-1, effects that were eliminated by eplerenone or by preventing EGFR transactivation [18]. Finally, the SFR was suppressed by MR blockade or by scavenging ROS, but it was unaffected by GR blockade or protein synthesis inhibition as shown in (Fig. **5C**). These results clearly suggest that MR activation is a necessary step in stretch-triggered mitochondrial ROS production that mediates the activation of redox-sensitive kinases upstream NHE-1, leading to the Anrep effect and also probably to cardiac hypertrophy development. However, a recent work *in vivo* in a transgenic mouse model of cardiomyocyte-targeted EGFR-deficient activation while confirmed the critical involvement of EGFR transactivation in A2-induced cardiac hypertrophy rejected it in cardiac hypertrophy due to MR activation.[19] The reason for this apparent discrepancy with our and others results are not clear at present. 

An alternative mechanism proposed by other investigators to explain the SFR involves the stretch-activated membrane channels (SACs). Myocardial stretch activates these non-selective cation channels, allowing Ca^2+^ and Na^+^ entry. The latter would then permit [Ca^2+^]_i_ increase via NCX, or even Ca^2+^ entry directly augmenting the Ca^2+^ transient amplitude and driving the SFR. A role for SACs in the SFR was suggested by Calaghan and White in 2004 [20], mainly as an addition to NHE1 activation in a 50-50% contribution manner. However, pharmacological strategies used to inhibit these channels were challenged based on the possible secondary actions of these compounds [21]. Recently, Ward *et al*. [22] reported in mouse trabeculae that opening of SACs appears to be the main contributor to the SFR after stretch. Specifically, transient receptor potential canonical channels (TRPC) 1 and 6 isoforms appear to be the suitable stretch-activated non-selective cation channels mediating the SFR. Interestingly, these authors inhibited the SFR using a peptide isolated from a spider venom (GsMTx-4) that seems to be specific to block SACs. At first glance, these results appear not to be in agreement with our proposal. However, we must consider the possibility that additional parallel or even in series pathways involving the aforementioned mechanisms could also exist.

Among our latest contributions to the understanding of the signalling pathway leading to the SFR the finding of a crucial role played by the MR was probably the most important. This may conceivably provide a mechanistic explanation to the strikingly beneficial effects of MR antagonism in patients with cardiac hypertrophy and failure [23-25], as it will be discussed latter in this review. 

In the following sections we will focus on this steroid hormone receptor, its downstream signalling pathway leading to NHE-1 activation and the potential link between MR-mediated NHE-1 activation and cardiovascular diseases. 

## ACTIVATION OF THE MR

The MR (or NR3C2), a member of the steroid/thyroid hormone receptor superfamily of ligand-inducible transcription factors is the receptor that mediates classic ALD effects. This receptor family includes the glucocorticoid (GR or NR3C1), thyroid (THRA, THRB), retinoic acid (RARA, RARB, RARC), and vitamin D receptors (VDR), as well as several orphan receptors. 

**Fig. (6):**
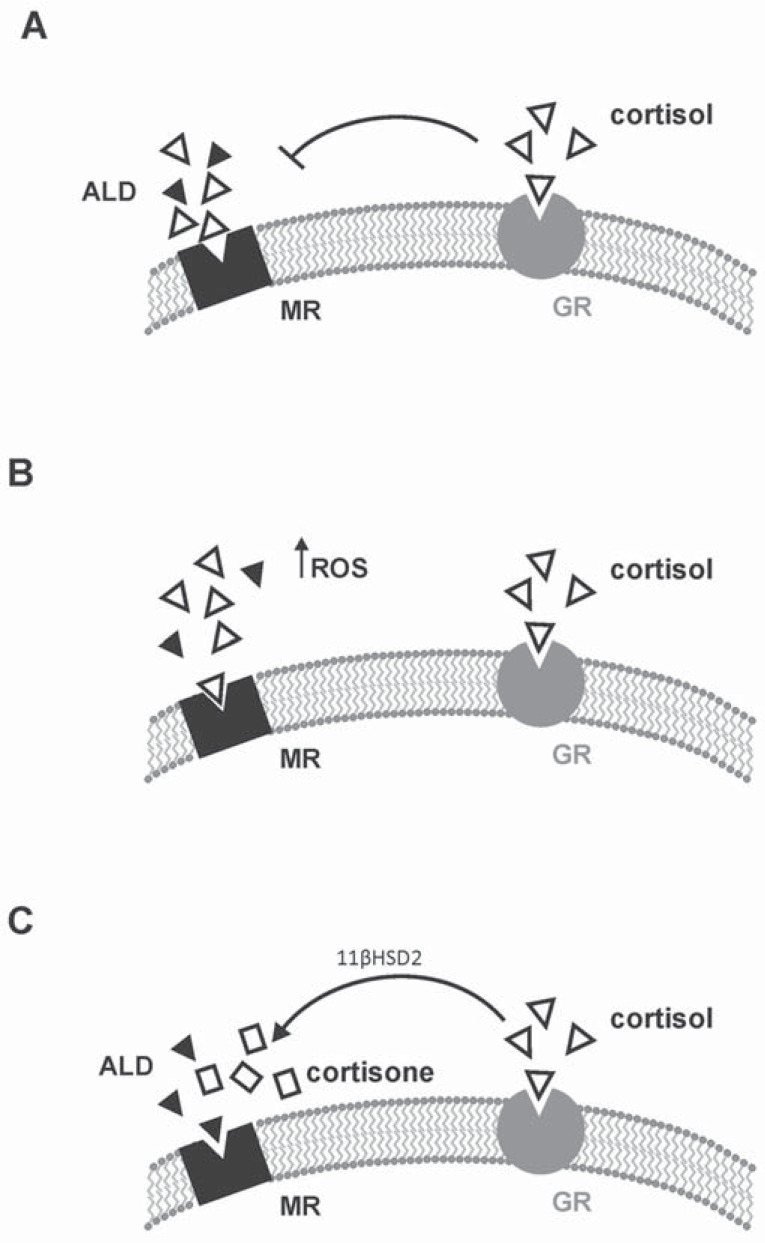
Schematic representation of the possible occupation of the mineralocorticoid receptor (MR) by physiological agonists. The MR has equivalent affinity for aldosterone (ALD) and cortisol, however circulating glucocorticoid levels are at least two orders of magnitude greater than those of ALD determining that MR are usually occupied, but not activated, by glucocorticoids (**Panel A**). Conversely, under pathologic conditions and increased oxidative stress glucocorticoids have been shown not only to occupy but also to activate the MR (**Panel B**).[30] In epithelial ALD-target cells the presence of the enzyme 11β-hydroxysteroid dehydrogenase (11βHSD2), that converts active glucocorticoids (e.g. cortisol) into receptor-inactive 11-keto analogs (e.g. cortisone), facilitates ALD occupancy and activation of the MR (**Panel C**).

Although the increase in ALD concentration constitutes the best recognized stimulus to MR, MR can also be activated in normal or even low-ALD states [26-28]. Moreover, the MR was shown to have equivalent high affinity for ALD, cortisol (corticosterone in rodents) and DOC [29]. This is a relevant fact since circulating glucocorticoid levels are at least two orders of magnitude greater than those of ALD determining that MR are usually occupied, but not activated, by glucocorticoids (Fig. **6A**). However, under pathologic conditions and increased oxidative stress glucocorticoids have been shown to activate the MR (Fig. **6B**) [30]. 

In epithelial ALD-target cells the presence of the enzyme 11β-hydroxysteroid dehydrogenase (11βHSD2), expressed at high levels, facilitates ALD occupancy and activation of the MR. The 11βHSD2 converts active glucocorticoids (e.g. cortisol) into receptor-inactive 11-keto analogs (e.g. cortisone), significantly reducing intracellular glucocorticoid levels (to ~10-fold those of ALD) [31-33] (Fig**. 6C**). 

In non-epithelial tissues such as the myocardium, hippocampus, vascular smooth muscle and adipose tissue the expression of 11βHSD2 is too low or inexistent to prevent cortisol to access the MRs in competition with much lower prevailing concentrations of ALD. Therefore, an unavoidable and yet unresolved question arises: how can aldosterone occupy and activate the MR, particularly in tissues with very low or no expression of the 11βHSD2? 

On the other hand the possibility exists of an ALD-independent MR activation*. *Among the putative mechanisms of ALD-independent MR activation in the cardiovascular system, several possibilities should be considered: (**1**) Glucocorticoid-mediated MR activation, especially under conditions of enhanced ROS production, as reported by Mihailidou *et al*. [30] as mentioned before (**2**) ligand-independent MR activation as the redox-sensitive Rac1-dependent activation proposed by Nagase *et al*. [34] (**3**) Direct MR phosphorylation independent of its own ligand, as proposed by Kato *et al*. [35] for the estrogen receptor. (**4**) Specific changes in MR conformation induced by strain, as proposed by Zou *et al*. [36] to explain AT1 receptor activation by mechanical stretch.

## NHE-1 STIMULATION BY MR ACTIVATION

**Fig. (7):**
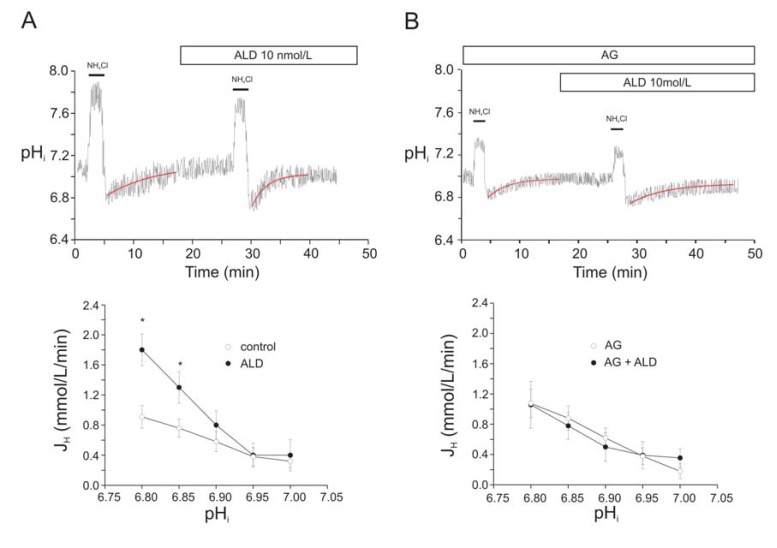
Aldosterone (ALD) activated the NHE-1. **Panel A, top:** representative traces of pH_i_ during the application of two consecutive ammonium pulses (20 mmol/L NH_4_Cl), in the absence (first pulse) and presence of 10 nmol/L ALD (second pulse). ALD was applied 10 min before the second pulse. **Panel A bottom:** average proton efflux J_H_, carried by the NHE-1, before (first pulses, closed circles) and after application of 10 nmol/L ALD (second pulses, open circles). * indicates p<0.05 vs. control. **Panel B top:** representative traces of pH_i_ during the application of two consecutive ammonium pulses (20 mmol/L NH_4_Cl), in the absence (first pulse) and presence of 10 nmol/L ALD (second pulse). AG1478 (AG, 1 µmol/L) was applied 10 min before the first pulse and maintained throughout the experiment. ALD was applied 10 min before the second pulse. **Panel B bottom:** average proton efflux J_H_, carried by the NHE-1, before (first pulses, open circles, n=4) and after application of 10 nmol/L ALD (second pulses, closed circles) in the continuous presence of 1 µmol/L AG1478. Modified from De Giusti *et al*. [43] with permission.

Fujisawa *et al*. [37] demonstrated that mineralocorticoid/salt-induced rat cardiac fibrosis and hypertrophy was prevented by the selective NHE-1 blocker cariporide. It has also been reported that ALD up-regulates the expression and function of NHE-1 [38-41] and that selective blockade of this ion exchanger prevents and/or reverts left ventricular hypertrophy in various animal models [42]. According to these data and in agreement with our previous results on the SFR, we have recently shown that ALD increases NHE-1 activity in rat ventricular myocytes through a non-genomic pathway (Fig. **7A**) [43].

As commented above, EGFR transactivation represents one of the signalling pathways triggered by ALD [44, 45]. It has been shown that the MR antagonist spironolactone reduces the EGFR mRNA synthesis after cerebral ischemia [46]. Accordingly, Grossmann *et al*. [45] reported that MR activation by ALD enhanced EGFR expression via an interaction with the EGFR promoter of vascular smooth muscle. In addition to these genomic effects, non-genomic actions of ALD involving EGFR transactivation have also been reported [11, 47]. Consistent with this evidence, we have recently shown that ALD enhances NHE-1 activity via transactivation of EGFR [43]. The stimulatory effect of this hormone on NHE-1 was prevented by blocking the EGFR with AG1478 (Fig. **7B**), and also by the inhibitor of the Src-kinase PP1 and the blocker of metalloproteinases MMPI [43]. These proteases release HB-EGF from its precursor, proHB-EGF. It has been reported that at least a fraction of the total amount of MR is bound to the sarcolemma, likely co-localized with the EGFR [14] and/or associated to caveolin-1 [48]. These data would explain the binding of ALD to the sarcolemmal fraction reported by Le Moellic *et al*. [49]. In addition, non genomic effects of ALD altering stimulation of a GPCR (GPR30) has been recently reported in vascular smooth muscle and endothelial cells [50].

## POTENTIAL LINK BETWEEN MR-MEDIATED NHE-1 ACTIVATION AND CARDIOVASCULAR DISEASE 

Cardiovascular disease and specially heart failure is one of the most important health problems in the world. As described above, cardiac hypertrophy and failure are triggered by intracellular signals that occur following myocardial stretch. Surprisingly, investigators working in the area of cardiac mechanics did not often extrapolate their early findings seen after stretch, such as the SFR, to the development of cardiac hypertrophy and/or failure. The reason for this could be that time frames in which these two phenomena occur are quite different. However, the long journey toward myocardial hypertrophy and failure begins with one step, and this first step may well be the autocrine/paracrine intracellular signalling pathway triggered by myocardial stretch as it was proposed in neonatal cardiac myocytes as well as in adult myocardium [3, 9, 10, 51, 52].

The pathophysiological role of ALD in the development of cardiovascular disease has long been considered to be due to sodium/water retention and hypertensive effect as a consequence of MR activation in renal tubular epithelial cells. However, recent accumulating evidence confirmed that the pathophysiological role of ALD is mediated not merely by its volume expansion/hypertensive effect, but also by its action through MR activation in non-epithelial cells of the cardiovascular system [23, 24, 53-55].

Clinical studies demonstrated the existence of a positive correlation between plasma ALD levels and cardiovascular damage [56, 57]. Moreover, despite complete vascular ACE inhibition plasma aldosterone levels are elevated in patients with heart failure [58]. Even the combination of ACE inhibition and angiotensin II antagonism only transiently reduces aldosterone plasma levels in patients with heart failure suggesting angiotensin II independent aldosterone production [59]. This phenomenon known as aldosterone escape represents a further reason to directly inhibit MR activation in heart failure. 

**Fig. (8):**
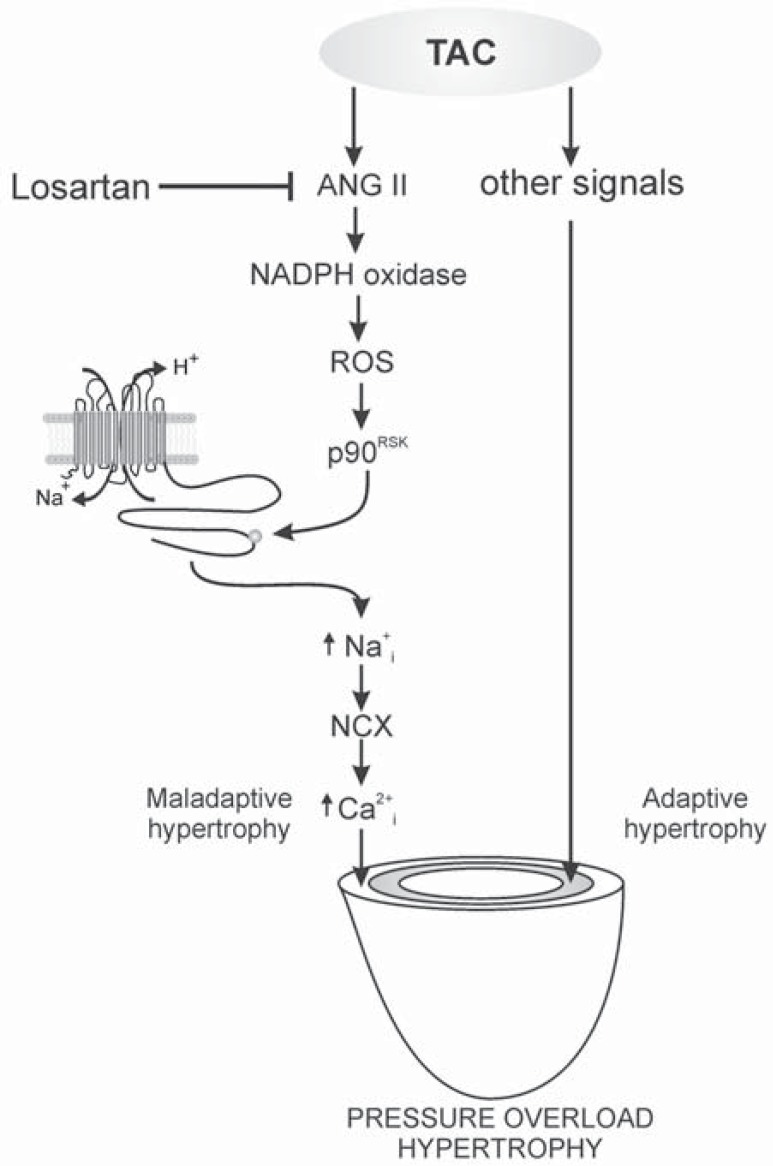
Schematic representation of the proposed signalling pathway involved in the prevention of cardiac hypertrophy by AT1 receptor blockade. In our scheme, the AT1 receptor-sensitive part of the transverse aortic constriction (TAC)-induced cardiac hypertrophy is maladaptive and related to redox-sensitive p90^RSK^ activation, NHE-1 phosphorylation/activation, increase in intracellular Na^+^ and the consequent increase in intracellular Ca^2+^ through the NCX. The increased Ca^2+^ concentration would then activate the calcineurin-NFAT signalling pathway responsible for triggering an abnormal cardiac growth. On the other hand, the same mechanical stimulus (stretch of cardiac muscle) may trigger other prohypertrophic signals intended to compensate for the increased wall stress ("adaptive hypertrophy"). The fact that *in vivo* TAC-overloaded myocardium presents increased NHE-1 activity (evidenced by the increase in NHE-1.and p90^RSK^ phosphorylation), supports that the stretch-induced activation of the exchanger detected in *in vitro* experiments persists after 7 weeks and it is able to be blunted by AT1 receptor blockade. Considering that A2 probably induces the activation of the myocardial MR, a contribution of the activation of this latter receptor to the development of inappropriate hypertrophy should be considered. Modified from Cingolani *et al*. [67] with permission.

Enhanced NHE-1 activity as a possible mechanism involved in cardiac hypertrophy and failure has been reported in the hypertrophic myocardium of adult spontaneously hypertensive rats (SHR) [60], in human ventricular myocytes from hearts with chronic end-stage heart failure [61], in a pressure-volume overload model of cardiac hypertrophy and failure in rabbits [62], in the hypertrophied heart of a type 2 diabetic rat model [63] and in neonatal rats [64]. Interestingly, Nakamura *et al*. [65] have recently demonstrated in vitro that NHE-1 hyperactivity is sufficient to generate calcium signals required for cardiac hypertrophy to take place. Although in vivo physiological data supporting the involvement of this mechanism in the transition to chronic cardiac hypertrophy and its consequences are scant, Baartscheer *et al*. [66] have shown in elegant experiments that long-term NHE-1 inhibition with cariporide in rabbits with combined pressure and volume overload cardiac hypertrophy and failure attenuated hypertrophy and decreased the previously augmented diastolic calcium without significant alteration of systolic calcium. Whether some early intracellular signals triggered by the autocrine/paracrine mechanism, (i.e; NHE-1 activation) persist over time, we recently found enhanced oxidative stress as well as increased phosphorylation of the redox-sensitive p90^RSK^ kinase and NHE-1 in a mouse model of cardiac hypertrophy and failure promoted by transverse aortic constriction (TAC). Selective AT1 receptors blockade with losartan prevented p90^RSK^ and NHE-1 activation and decreased hypertrophy development, preserving contractility in spite of a higher workload (Fig. **8**) [67].

## FACTS AND PERSPECTIVES IN HEART FAILURE TREATMENT 

Current treatment against cardiac failure is mainly based on inhibition of hormones (A2, ALD, catecholamines). Despite the term “ALD inhibition” has been widespread used, this is often misleading and should be replaced by MR antagonism, mainly because ALD is not the only agonist binding to and activating MR as mentioned before [30]. Although several studies have demonstrated the important benefits of MR antagonists in heart failure, their clinical use remains lower than expected and the exact mechanism of the beneficial effect is still unknown.

Among MR inhibitors, spironolactone was the first marketed compound in the early 1960s, and although proved to be clinically useful, it also showed tolerability problems. Nevertheless, it was the only compound approved to be used in the RALES in patients with severe heart failure (Class III-IV NYHA). The trial was terminated prematurely due to an interim analysis revealing a ~30% reduction in the relative risk of death in spironolactone-treated patients [24]. There was an equal and impressive reduction in hospitalization for cardiac reasons. Later on, more specific MR antagonists were developed and clinically tested. The EPHESUS clinical trial was performed on 6642 patients with acute myocardial infarction complicated with left ventricular systolic dysfunction. Treatment started 3 to 14 days after myocardial infarction and was maintained during 16 months. All cause mortality decreased by ~15% and sudden cardiac death by ~21 % in the eplerenone-treated arm [23]. 

Contrasting with the two above-mentioned clinical trials, the recently published EMPHASIS [25] was carried out on patients with less severe heart failure. This study enrolled 2737 patients with heart failure class II and III of the NYHA and left ventricular ejection fraction of no more than 35 %. The trial was stopped prematurely after a median follow-up of 21 months, due to the excess of benefit in reducing the risk of cardiovascular death or hospitalization for heart failure, obtained by anti-aldosteronic therapy with eplerenone, which was then extended to both arms of the trail.

Although clinical evidence undoubtedly showed beneficial effects of treating heart failure patients with MR blockers, the mechanisms by which MR antagonism provide cardiovascular protection are not completely understood. In this regard, our own results assigning a crucial role for MR activation as an early hypertrophic signal triggered by myocardial stretch encouraged us to suggest that prevention of oxidative stress and NHE-1 activation should be considered as a potential key factor for the salutary effects of ALD antagonism in humans.
